# Increased expression of CD25, CD83, and CD86, and secretion of IL-12, IL-23, and IL-10 by human dendritic cells incubated in the presence of Toll-like receptor 2 ligands and *Giardia duodenalis*

**DOI:** 10.1186/1756-3305-6-317

**Published:** 2013-11-04

**Authors:** Janine Obendorf, Pablo Renner Viveros, Michael Fehlings, Christian Klotz, Toni Aebischer, Ralf Ignatius

**Affiliations:** 1 Institute of Tropical Medicine and International Health, Charité – Universitätsmedizin Berlin, Spandauer Damm 130, Berlin 14050, Germany; 2 Mycotic and Parasitic Agents and Mycobacteria, Department of Infectious Diseases, Robert Koch-Institute, Nordufer 20, Berlin 13353, Germany

**Keywords:** Dendritic cells, *Giardia duodenalis*, Toll-like receptors, CD25, CD83, CD86, IL-12, IL-23, IL-10

## Abstract

**Background:**

Effects of *Giardia duodenalis* on dendritic cell (DC) functions may contribute to the pathogenesis of chronic giardiasis. *G. duodenalis* lysate has been shown to inhibit the activation of murine DCs through the ligands of various Toll-like receptors (TLRs), including TLR2 and TLR4. Our study aimed at translating these findings to human DCs.

**Findings:**

As described previously for murine DCs, also human DCs were only weakly activated by the parasite itself. LPS-stimulated DCs incubated in the presence of *G. duodenalis* lysate produced less IL-12/23p40 (p = 0.002), IL-12p70 (p = 0.011), and IL-23 (p = 0.004), but more IL-10 (p = 0.006) than cells incubated in the absence of the parasite. Concomitantly, the expression of CD25, CD83, CD86, and HLA-DR was reduced on *G. duodenalis-*incubated DCs as compared to control cells. In contrast, human DCs stimulated through TLR2 in combination with TLR1 or TLR6 and *G. duodenalis* lysate secreted significantly more IL-12/23p40 (p = 0.006), IL-23 (p = 0.002), and IL-10 (p = 0.014) than cells stimulated through TLR2 ligands alone. Ligands for TLR2/TLR1 or TLR2/TLR6 also induced enhanced extracellular expression of CD25, CD83, and CD86 (p < 0.05).

**Conclusions:**

In contrast to murine DCs, human DCs incubated in the presence of *G. duodenalis* and stimulated through TLR2 show increased activation as compared to cells incubated in the absence of the parasite. Thus, TLR2 ligands, e.g., delivered by probiotic lactobacilli, might be beneficial in human giardiasis through an adjuvant effect on the induction of cellular immune responses against *G. duodenalis*.

## Findings

### Background

*Giardia duodenalis* is the most frequent parasitic agent of gastroenteritis worldwide and has been targeted as part of the WHO “Neglected Disease Initiative” since 2006 [1]. In East Africa, for example, we and others have observed prevalences of >60% in children younger than five years [2,3]. Notably, chronic (or recurrent) giardiasis in children has been associated with malnutrition, wasting, and stunting as well as reduced cognitive functions at a later age [4].

The pathogenesis of chronic giardiasis is poorly understood and both parasitic and host factors might be involved. The parasite is non-invasive but stays in the intestinal lumen where it adheres to epithelial cells. Since CD4^+^ T helper cells are involved in protection against *G. duodenalis*[5], chronic infection might result from insufficient antigen presentation that does not lead to the development of protective cellular immunity. Dendritic cells (DCs) might be critically involved in the induction of anti-*Giardia* immune responses as they are able to reach with their cellular protrusions into the gut lumen and sample antigen [6]. Following antigen-uptake, DCs may process and present specific peptides to naïve T lymphocytes, and DCs activated through ligands of pattern-recognition receptors (PRRs), e.g., Toll-like receptors (TLRs), C-type lectins, and nucleotide-binding oligomerization domain (NOD)-like receptors (NLRs), are essential for the induction of specific cellular immunity [7]. A recently published study with murine DCs has shown that *G. duodenalis* lysate may interfere with the activation of DCs through TLR2 or TLR4 *in vitro*[8]. Signals through both receptors might be relevant for the induction of anti-*Giardia* immune responses because *G. duodenalis* by itself only weakly activates murine DCs [8]. *In vivo*, bacteria belonging to the gut flora might deliver the required stimuli to *G. duodenalis*-processing DCs since TLR2 may recognize lipoproteins derived from Gram-positive while TLR4 binds LPS of Gram-negative bacteria [9].

The present study aimed at translating the previous findings from murine to human DCs. To monitor DC activation, we focused on the expression of typical surface markers and cytokine production [10]. Regarding cytokines, we analyzed the secretion of members of the IL-12 family. IL-12 is a pro-inflammatory cytokine, which consists in its biologically active form, IL-12p70, of a light chain (IL-12p35) and a heavy chain (IL-12p40), and favors the differentiation of Th1 cells [11]. IL-12p40 (together with a p19 subunit) may also be part of IL-23, which drives the development of T helper cells that secrete IL-17 (Th17) [12]. In addition, we analyzed the secretion of the anti-inflammatory cytokine, IL-10.

## Methods

### Preparation of *G. duodenalis* lysate

*G. duodenalis* strain WB-C6 (ATCC 50803) trophozoites were propagated in TYI-S-33 medium as previously described [13]. Parasites were harvested and washed by centrifugation through PBS. Pellets were resuspended in PBS, and the parasites counted and diluted to a concentration of 10^8^ parasites/ml. Lysates were prepared by subjecting the suspensions in three consecutive freezing-thawing cycles.

### Generation of human DCs

DCs were generated as described previously [14]. Briefly, peripheral blood mononuclear cells were isolated from buffy coats obtained from healthy donors (German Red Cross, Berlin). CD14^+^ monocytes were separated with magnetic microbeads (Miltenyi Biotec, Bergisch-Gladbach, Germany) and cultured at 3x10^6^ cells/well in 6-well cell culture dishes (Nunc, Roskilde, Denmark) for six days in medium consisting of RPMI 1640 supplemented with 2 mM L-glutamine, 10 mM HEPES, penicillin (1,000 U/ml)-streptomycin (1,000 μg/ml) (all from Gibco, Invitro-gen, Karlsruhe, Germany), 50 μM 2-mercaptoethanol (Sigma, Taufkirchen, Germany), 10% fetal calf serum (Biochrom, Berlin, Germany), 1000 U/ml human rGM-CSF (sargramostim, Leukine®, Berlex, Richmond, CA, USA), and 100 U/ml human rIL-4 (R&D Systems, Wiesbaden-Nordenstadt, Germany). On days 2 and 4, fresh medium and cytokines were added to the wells.

### Incubation of human DCs with *G. duodenalis* lysate and TLR ligands

On day 6, DCs were harvested and transferred to 96-well round-bottom trays (Nunc) at 10^5^ cells/well. The TLR stimuli Pam2CSK4 and Pam3CSK4 (100 ng/ml; Invivogen, Toulouse, France) or LPS (1 μg/ml; derived from *Escherichia coli* 0111:B4, Sigma) were added and the cells incubated in the presence or absence of *G. duodenalis* lysate (equivalent to five trophozoites/cell). Immature cells kept in medium were equally incubated in the presence or absence of *G. duodenalis* lysate.

### Flow cytometric analyses

The phenotype of DCs was monitored by flow cytometry with PE- or FITC-labeled anti-human mAbs against HLA-DR, CD14, CD25, CD86 (all BD Pharmingen), CD83 (Caltag Laboratories, Hamburg, Germany), or the appropriate isotype controls, as previously described [14]. Cells were fixed with 10% formalin/PBS (v/v) before analysis on a FACScan® cytometer with CELLQuest®Pro software (BD Pharmingen). Percentages of positive cells and median fluorescence intensities (MFIs) were calculated.

### Analysis of cytokine secretion by DCs

Cell-free supernatants of cell cultures were harvested after 48 h and stored at -80°C until analysis by sandwich ELISAs for IL-12p40, IL-12p70, and IL-10 (all U-CyTech, Utrecht, The Netherlands), or IL-23 (eBioscience). Samples with known cytokine concentrations provided by the manufacturers of the ELISA kits were included in each analysis and used to create standard curves. Only values within the linear section of these curves were considered. The level of detection was 5 pg/ml for all assays.

### Statistical analyses

Data were analyzed statistically with the nonparametric Wilcoxon matched-pairs signed rank test using GraphPad Prism version 6.0a for Mac OS X. Differences were considered statistically significant for p < 0.05.

## Results

We incubated human immature monocyte-derived DCs in the presence or absence of *G. duodenalis* lysate with the TLR4 ligand LPS or with ligands binding to TLR2/TLR1 or TLR2/TLR6, i.e., Pam3CSK4 and Pam2CSK4, respectively, since TLR2 is expressed together with either TLR1 or TLR6 [9]. Control cells were kept in the absence of TLR ligands. Two days later, supernatants were collected and the cells harvested.

Flow cytometry revealed that similar to murine DCs, *G. duodenalis* lysate by itself only weakly activated human DCs (data not shown). On LPS-activated DCs, it reduced the expression of CD25 and CD83, typical DC surface markers indicative of cellular activation, and of CD86 and HLA-DR, which are involved in antigen-presentation (Table 1). Similarly, the MFIs for CD83 and CD86 were reduced on *G. duodenalis*-incubated DCs as compared to control cells (data not shown). In contrast, among DCs stimulated through either TLR2/TLR1 or TLR2/TLR6 and incubated in the presence of *G. duodenalis* lysate, significantly more cells expressed CD25, CD83, and CD86 than in DC populations incubated in the absence of the parasite-derived stimuli (Table 1). These larger numbers of CD25 and CD83 positive cells also correlated with significantly higher MFIs for these molecules (data not shown).

**Table 1:** **DC activation markers expressed by human DCs incubated in the presence of ligands for TLR4, TLR2/TLR1, or TLR2/TLR6, and in the presence or absence of *G. duodenalis *** **lysate**

**Molecule**	**LPS**	**LPS +** ***Gd***	**Pam3CSK**	**Pam3CSK +** ***Gd***	**Pam2CSK4**	**Pam2CSK4 +** ***Gd***
CD25	84 [73, 90]^#^	82 [66, 88]*	8 [3, 21]	20 [7, 30]*	10 [3, 27]	21 [9, 35]*
CD83	90 [85, 95]	84 [80, 93]*	18 [12, 51]	35 [22, 59]*	23 [15, 51]	39 [24, 63]*
CD86	98 [97, 99]	96 [93, 97]*	51 [48, 61]	67 [59, 79]*	56 [48, 71]	70 [61, 86]*
HLA-DR	99 [98, 99]	97 [95, 98]*	88 [84, 97]	90 [86, 97]	88 [84, 96]	92 [84, 97]

^#^Median percentage of positive cells (25% and 75% percentiles given in square brackets) obtained with cells from ten donors, except for CD86, which could be analyzed for eight donors only (data of isotype controls were subtracted).

*p < 0.05 compared to DCs incubated in the presence of LPS, Pam3CSK, or Pam2CSK4 but in the absence of *G. duodenalis* (Wilcoxon matched-pairs signed rank test).

**Figure 1:**
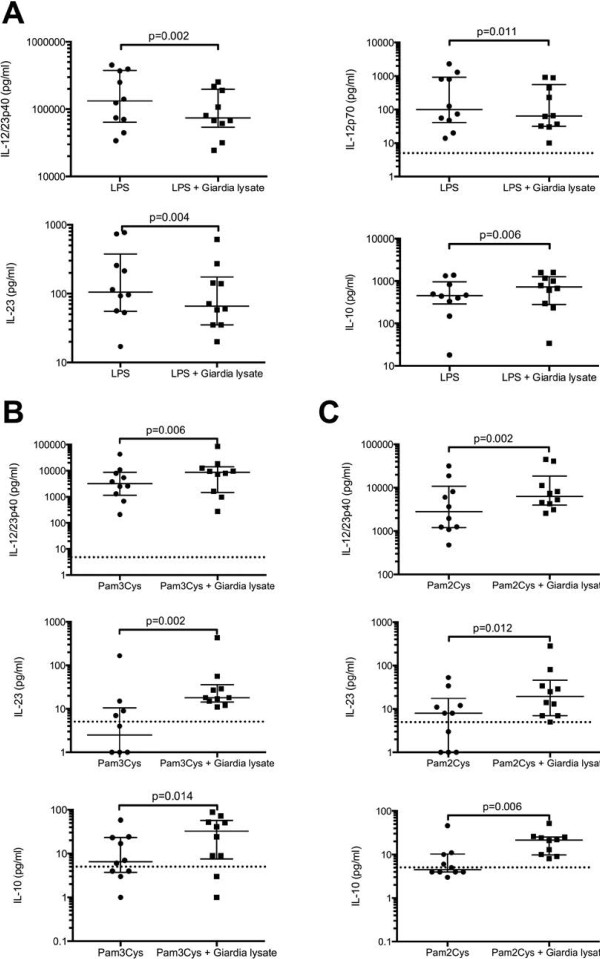
**TLR2 ligands but not LPS augment the secretion of IL-12 cytokines by human DCs incubated in the presence of *G. duodenalis*.** Immature DCs were incubated with **(A)** LPS (1 μg/ml), **(B)** Pam3CSK4 (100 ng/ml), or **(C)** Pam2CSK4 (100 ng/ml) in the presence or absence of *G. duodenalis* lysate (equivalent to five trophozoites/cell). Two days later, cell-free supernatants were collected, stored at -80°C, and the cytokine concentrations determined by ELISA. Median plus 25% and 75% percentiles for cells from ten donors. Dotted horizontal lines indicate the level of detection of the ELISA assays (5 pg/ml).

We next determined the concentrations of IL-12/23p40, IL-12p70, IL-23, and IL-10 in all supernatants and again observed distinct effects of *G. duodenalis* lysate dependent on the TLR agonist involved. When cells were activated through TLR4, *G. duodenalis* significantly reduced the production of IL-12/23p40, IL-12p70, and IL-23 while the IL-10 secretion was enhanced (Figure 1). In contrast to these findings and those previously obtained with murine DCs, exposure to *G. duodenalis* lysate enhanced the secretion of IL-12/23p40, IL-23, and IL-10 by DCs activated through TLR2 – although the cells produced comparably less cytokines than LPS-stimulated DCs (Figure 1). The IL-12p70 concentrations of TLR2-activated DCs were below the level of detection for most samples, irrespective of the presence or absence of *G. duodenalis* lysate in the cell cultures. As noted before for LPS-stimulated human monocytes [15], we also observed inter-individual differences, i.e., low and high responders, in the cytokine responses upon TLR-ligation of the DCs. The discordant values, however, did not affect the statistical analyses of the data.

Thus, while we could translate the previous murine data regarding the inhibition of LPS-stimulated DCs through *G. duodenalis* to the human cell system, we observed the opposite for DCs stimulated through TLR2. Exposure of TLR2-activated DCs to *G. duodenalis* considerably enhanced the expression of crucial surface molecules and the secretion of cytokines belonging to the IL-12 family.

## Conclusion

In contrast to TLR4 ligands and in contrast to murine DCs, TLR2 ligands may augment the antigen-presenting functions of human DCs in giardiasis. Although the LPS-induced cytokine responses were comparably stronger than those induced by the TLR2 ligands, which might be due to the concentrations of the stimuli used, we feel that the data together, i.e., opposite results for cytokine secretion and also surface molecule expression for the stimuli tested, may argue for an additive effect of TLR2 and *G. duodenalis* on human DC functions. In contrast, *G. duodenalis* apparently may interfere with immune responses induced by human DCs activated through TLR4 agonists.

Probiotic *Lactobacillus* spp. are recognized by human DCs through TLR2 and NOD2 [16], and may induce – dependent on TLR2 – the secretion of IL-12, IL-10, and type I interferons as shown for murine DCs [17]. In a Gerbil model, interestingly, lactobacilli interfere with *G. duodenalis* infection *in vivo*[18], and this effect could partly depend on TLR2 signaling. Although this remains to be investigated, our demonstration that *G. duodenalis* can augment TLR2-mediated DC function suggests that TLR2 ligands might be of considerable interest in the management of clinical giardiasis. Since an anti-giardial effect by *Lactobacillus* spp. on murine giardiasis is also evident when the bacteria are administered together with anti-giardial drugs [19] or in malnourished animals [20], clinical studies are warranted in which the potential benefit of lactobacilli in the treatment of human giardiasis patients may be evaluated as a combination of bacteria with standard therapy, e.g., metronidazole or tinidazole, but also in therapy-refractory chronic giardiasis.

## Competing interests

The authors declare that they have no competing interests.

## Authors’ contributions

JO and PRV performed the experiments, analyzed the data, and wrote the manuscript; MF analyzed the data and wrote the manuscript; CK and TA prepared the *G. duodenalis* lysate, designed the study, and wrote the manuscript; RI designed and supervised the study and wrote the manuscript. All authors have approved the final version of the manuscript.
